# The importance of data from the atomic bomb survivors for international radiological protection

**DOI:** 10.1093/carcin/bgaf062

**Published:** 2025-10-27

**Authors:** Richard Wakeford, Dominique Laurier

**Affiliations:** Centre for Occupational and Environmental Health, The University of Manchester, Manchester M13 9PL, United Kingdom; French Nuclear Safety and Radiation Protection Authority (ASNR), Directorate of Health Research and Expertise, Fontenay aux Roses F-92262, France

**Keywords:** atomic bomb survivors, ionising radiation, epidemiology, health effects, radiological protection

## Abstract

Epidemiological studies using data from the lifetime experience of the survivors of the atomic bombings of Japan have been fundamental in shaping the international system of radiological protection since a clear excess risk of, first leukaemia and then other cancers, became apparent in the 1950s and 1960s. Cancer mortality and incidence data have been collected, collated and analysed by the Radiation Effects Research Foundation (RERF, and its predecessor, the Atomic Bomb Casualty Commission, ABCC) and the risk estimates so produced have been used subsequently by the International Commission on Radiological Protection (ICRP) to provide the main technical basis of its general recommendations, from its 1965 Recommendations to the latest 2007 Recommendations. As the database has grown with increasing follow-up of the survivors, together with continued refinement of the dosimetry system, ABCC/RERF analyses have become more sophisticated, permitting knowledge of the variation of risks for a growing number of specific types of cancer, as modified by sex, age-at-exposure and time-since-exposure/attained age. A growing database of non-cancer effects, such as diseases of the circulatory system and eye lens opacities, is also providing risk estimates. Even now, 80 years after the bombings, the survivors provide important information on risks decades after exposure at a young age. There is little doubt that recent RERF studies of cancer and non-cancer diseases in the survivors will continue to steer the ICRP in its progress towards the next Recommendations.

## Graphical Abstract

**Graphical Abstract:**
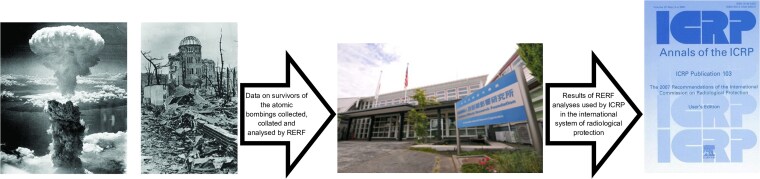

## 1. Introduction

The international system of protection against adverse health effects consequent to exposure to ionising radiation has been established by the International Commission on Radiological Protection (ICRP), and the latest version of this system is laid out in the 2007 Recommendations of the ICRP [1]. The roots of ICRP lie in the International X-ray and Radium Protection Committee (IXRPC), which was established in 1928 by the Second International Congress of Radiology meeting in Stockholm [2–5]; the name of the IXRPC was changed to ICRP in 1950.

Before the Second World War, the IXRPC concerned itself with protection against harmful tissue reactions (deterministic effects) produced by high (and for some effects, moderate) and mainly localized doses received at a high dose rate, primarily in the context of exposures incurred in the practice of medicine, and set dose limits to prevent such effects. However, during the 1940s, interest grew in the possible long-term carcinogenic effects of protracted exposures to radiation, with epidemiological studies reporting excesses of leukaemia among radiologists, who had presumably accumulated substantial doses of radiation but over reasonably long periods, although recorded doses were generally lacking [6–9].

But it was the atomic bombings of Hiroshima and Nagasaki in August 1945 that generated a step-change in attitudes towards radiological protection against exposure to radiation. After the atomic bombings, radiological protection addressed exposure of the general public (not only patients) rather than focussing upon occupational exposure of radiologists (and other workers). The cohort of Japanese atomic bomb survivors became the foundation of the assessment of risks related to exposure to radiation, and thus at the heart of the evolution of the radiological protection system. In particular, it was these data that, over decades, led to the quantification of the long-term risk of cancer at low-to-moderate doses. The bomb survivor data have also provided information on the long-term risks of non-malignant diseases, such as diseases of the circulatory system and cataracts. Even today, 80 years after the Hiroshima and Nagasaki bombings, updates of the data continue to play a major role in consolidating the radiological protection system.

Over time, several studies were implemented and conducted using data from the Japanese atomic bomb survivors, initially by the Atomic Bomb Casualty Commission (ABCC) and after 1975 by its successor, the Radiation Effects Research Foundation (RERF). The most famous is the Life Span Study (LSS), a cohort study of a sample of ∼120 000 Japanese persons in the two cities either present during the bombings or unexposed immigrants, initiated in 1950 jointly by the Japanese National Institute of Health (JNIH) and ABCC to investigate the late health effects (mortality and cancer incidence) of irradiation during the bombings. A series of LSS Reports has been published by ABCC/RERF from 1962 onwards, reporting mortality results from the continuing follow-up of the cohort. A cohort of those who were exposed in their mother’s womb (exposure *in utero*) was also constructed. Further, a cohort of offspring of survivors, who were conceived after parental exposure (the first filial, F1, generation), was also launched to investigate potential hereditary effects due to exposure of parental germ cells. In addition, the Adult Health Study (AHS) was established as a subset of the LSS members, a health examination programme at ABCC/RERF based on clinical investigations. Biosamples provided by the AHS participants at the time of health examinations have been frozen and stored and will be of substantial value to future biomedical research [10].

The purpose of this paper is to trace the history of findings from the follow-up of survivors of the atomic bombings of Hiroshima and Nagasaki, and their offspring, to show the substantial impact on the development of the radiological protection system that the results from the ABCC/RERF studies have had. We focus on the health effects attributable to ionising radiation, but it should not be forgotten that the Japanese data have also influenced the dosimetric and ethical aspects of radiological protection.

## 2. Hereditary effects

Initially, the principal concern following the atomic bombings of Japan was potential hereditary effects in the offspring of irradiated survivors, with initiation of the genetics programme in Hiroshima and Nagasaki under the auspices of the ABCC [11]. It was known from the experiments of H. J. Muller that x-rays could produce mutations in the germ cells of fruit flies resulting in adverse hereditary effects in offspring [12, 13], so a programme of investigation to detect hereditary genetic effects in the offspring of Japanese survivors exposed before the conception of their children got underway in the late-1940s. However, Muller [14] opined that on the basis of existing knowledge it was unlikely that the effects of an irradiation-induced increase in mutations would be detectable, and it soon became apparent from the Japanese study that an excess risk of hereditary effects was not discernible in the offspring of exposed survivors [15–18]. The conclusion was reached in a collection of papers reporting the results of the study published in 1956 [11]:

‘…we can say of the present study…*that conspicuous genetic effects of the atomic bombs…have in fact not been demonstrated* [authors’ emphasis]’.

Muller [19] (a member of the ICRP Main Commission during 1950–65) reiterated his view that irradiation-related hereditary effects in the offspring of the atomic bomb survivors were unlikely to be detectable based on contemporary knowledge, because the numbers of offspring and parental doses were insufficiently high for a ‘signal’ of exposure to be discerned above the background ‘noise’ of effects, a point that had already made by Neel *et al*. [15]. Even so, the absence of detectable irradiation-related hereditary effects was reassuring.

In its 1958 Report [20], the United Nations Scientific Committee on the Effects of Atomic Radiation (UNSCEAR) concluded that on the basis of the study of the offspring of the Japanese atomic bomb survivors [11] it was ‘improbable that the representative doubling dose for human genes irradiated in gonial cells lies below 10 rad [100 mGy]’.

A comprehensive assessment of the hereditary risks of irradiation was made in the mid-1960s in ICRP Publication 8 [21, 22]. However, the inability to reliably quantify risks in exposed humans, primarily the atomic bomb survivors, meant that risk estimates for hereditary effects remained founded in the results of animal experiments.

The follow-up of the F1 cohort of ∼58 600 singleton children with at least one parent who was present in Hiroshima or Nagasaki at the times of the atomic bombings (and ∼16 700 children, neither of whose parents were in the two cities at the times of the bombings), born during 1946–84, has continued with the findings of follow-up to end-2009 reported by Grant *et al*. [23]. No discernible effects on mortality rates were found [23].

Recently, Yamada *et al*. [24] reanalysed the early data on pregnancy outcomes among children born to atomic bomb survivors using updated estimates of parental gonadal doses and refined analytical methods. A positive association was reported for major congenital malformations and perinatal deaths, but statistical significance (0.01 < *P* < .05) was observed only for conjoint dose (i.e. the sum of the gonadal doses of the parents) and perinatal deaths within 14 days. The authors also noted that confounding factors such as poverty, which was increased in areas close to the hypocentres in the years following the end of the war, need to be borne in mind when interpreting these results.

A review of studies of hereditary effects in the offspring of Japanese atomic bomb survivors is provided by Nakamura *et al*. in a companion paper in this Special Issue [25]. Among the studies currently planned at RERF is the Trio Genome Study, the whole genome sequencing of exposed parents and their offspring that should provide substantial evidence on the degree to which irradiation-induced damage is heritable [25].

The subject of how hereditary effects should be considered in the system of radiological protection is being addressed by ICRP Task Group 121 (https://www.icrp.org/icrp_group.asp?id=189). The way in which the ICRP has taken account of the risk of hereditary effects over the years has been reviewed by Amrenova *et al*. [26]—in essence, the treatment of hereditary risk by ICRP continues to be based in the results of experiments with laboratory animals plus knowledge of hereditary mechanisms in humans.

As Jordan has noted [27], the absence of detectable irradiation-related hereditary effects in tens of thousands of children of atomic bomb survivors, while it has been recognized for decades that this is not unexpected, is nonetheless a stark reminder that such effects are not at the high levels frequently perceived by laypeople. This is the principal value of the F1 cohort: these epidemiological data contribute significantly to the evidence that irradiation-related hereditary effects in humans, which are presumed to exist, are small and difficult to determine reliably [28].

## 3. Somatic effects

### 3.1. Initial findings

Within a few years of the atomic bombings certain adverse health effects became manifest in the survivors. After the publication in 1947/1948 of the findings of autopsy [29, 30] and clinical [31] studies of ocular damage following irradiation during the bombings, in 1949 it was reported that ten cases of cataracts in survivors (all of whom suffered ‘profound epilation of the head’) were believed to have been caused by irradiation of the lenses of their eyes during the two explosions [32]. Then, in 1952, the results of a study of congenital malformations in 205 children exposed in Hiroshima during the first half of intrauterine life were published [33]. The study found that 7 of the 11 children who were within 1200 m of the hypocentre had microcephaly with mental retardation, while none of the other 194 children were so affected. Both of these effects were the subject of continuing studies (see below).

### 3.2. Leukaemia

In the first half of the 1950s, reports of excess cases of leukaemia in the heavily exposed survivors were published [34–37]. These findings were extended and summarized in later publications in the late 1950s and early 1960s [38–40], with the conclusions [39, 40] that

- the risk of leukaemia was increased at doses of 0.5–1.0 Gy and above;
- the relationship between dose and leukaemia incidence is compatible with a linear model;
- when extrapolated to zero dose the curve intersects the expected background incidence, but the data were compatible with a variety of models, including a threshold;
- data for the period 1945–58 suggested an average irradiation-associated incidence rate of 1–2 cases per million person-years per cGy, although this is a very approximate estimate;
- the distribution of leukaemia by type did not appear to be markedly altered by exposure;
- the first cases were noted in 1947, the excess peaked in 1950–1952 but was still apparent in 1958;
- the excess incidence rate was greatest in those exposed at <10 years of age.

In 1960, the first report of the Hiroshima cancer registry was published [41], which indicated a notable increase in the incidence of leukaemia during 1957–8 among heavily exposed survivors. Subsequent registration data from Hiroshima and Nagasaki for 1957–9 confirmed the high rate of leukaemia incidence among survivors within 1500 m of the hypocentres [21, 22].

The leukaemia findings for the Japanese atomic bomb survivors as summarized in ICRP Publication 8 [21, 22] (see below) fed into the 1965 Recommendations of the ICRP [42], and these results were central to the understanding of the irradiation-related risk of leukaemia that formed a major part of the basis of radiological protection at that time. Based on the experience of the atomic bomb survivors [40], ICRP Publication 8 [21, 22], assuming a linear dose-response, assessed the leukaemia risk as 1 or 2 cases per million persons per year per 10 mGy over a period of 10–20 years following exposure.

Subsequent reports of leukaemia incidence and mortality among the bomb survivors have updated and expanded the risk estimates used in radiological protection (see below).

### 3.3. Cancers other than leukaemia

The first report of the Hiroshima cancer registry indicated not only a raised incidence rate of leukaemia during 1957–8 but also of solid cancer among the heavily exposed survivors [41]. LSS Report 1, published in 1962 [43], confirmed excess deaths from leukaemia but otherwise did not find evidence of an effect of irradiation on mortality rates. Nonetheless, subsequent cancer registration data from Hiroshima and Nagasaki for 1957–9 confirmed the raised incidence rate for solid cancer among survivors within 1500 m of the hypocentres, although much less pronounced than that for leukaemia [21, 22]. The raised levels of solid cancer were confirmed by LSS Reports 2 [44] and 3 [45], published in 1964 and 1965, respectively, which found an excess of deaths from solid cancer among heavily exposed survivors with a relative increase less than that for leukaemia.

Indications of an increase in thyroid cancer in the atomic bomb survivors were apparent in the early 1960s [46, 47], and confirmed a few years later [48] when the importance of a young age-at-exposure was identified.

In 1964, ICRP set up a Task Group on the Evaluation of Risks from Radiation, under Committee 1 of the Commission, ‘to consider the extent to which the magnitude of somatic and genetic risks associated with exposure to radiation can be evaluated’. The resulting report, ICRP Publication 8 [21, 22], was presented to Committee 1 in April 1965, and is a comprehensive and impressive review of the contemporary evidence for adverse health effects subsequent to radiation exposure. The report considered low levels of exposure to radiation, ‘regarded as doses of <50 rads [500 mGy] received acutely at high intensity, or multiple doses, totalling <10 rads [100 mGy] a year, received irrespective of dose rate’.

For cancer, evidence from the atomic bomb survivors, ankylosing spondylitis patients and antenatal medical x-ray examinations was assessed. The excess number of all other fatal malignancies appeared to be comparable to that of leukaemia, but since the baseline rate is considerably greater the percentage increase above the background is correspondingly smaller than with leukaemia. The numbers of thyroid cancers are compatible with expectations from patients receiving radiotherapy in childhood.

The Task Group findings contributed significantly to the 1965 Recommendations of the ICRP, Publication 9 [42], which marked a step-change in the approach of ICRP to radiological protection against low-level exposures. The assumption was made that for cancer ‘the risk of radiation injury is directly proportional to the accumulated dose’ and ‘down to the lowest levels of dose’. In other words, the ICRP 1965 Recommendations adopted what would now be termed the linear no-threshold dose-response model for the purposes of radiological protection against irradiation-related cancer. The findings of the ABCC studies of the survivors of the atomic bombings of Hiroshima and Nagasaki, as set out in ICRP Publication 8 [21, 22], played a pivotal role in this major change in approach.

LSS Reports 4 and 5 were published in 1971 [49] and 1972 [50], respectively, and used the Tentative 1965 Dosimetry (T65D) system; previous LSS Reports had used the Tentative 1957 Dosimetry (T57D) system. Reports 4 and 5 presented a dose-related excess of solid cancer mortality and were published ahead of the ICRP 1977 Recommendations, ICRP Publication 26 [51]. Largely on the basis of the LSS results, ICRP concluded that the sex- and age-averaged risk of cancer mortality was about 1% per Sv, and this risk estimate played a significant role in the Commission’s deliberations on setting dose limits [51].

LSS Reports 6 and 7 were published in 1978 [52] and 1982/1983 [53–55], respectively, followed by Reports 8 and 9 in 1987 [56] and 1989–92 [57–59], respectively. LSS Report 9 was the first to use the Dosimetry System 1986 (DS86) dose estimates and considered the changes to cancer mortality risk estimates brought about by the DS86 doses; Preston and Pierce [60] also examined these changes. The UNSCEAR 1988 Report [61] assessed the evidence that had been published in these recent LSS reports, and concluded that the estimated lifetime risk of cancer mortality was in the range 4%–11% per Sv. However, the Committee agreed that this risk estimate should be reduced by a factor in the range 2–10 for risks arising from exposure to low doses or at low dose rates.

The ICRP 1990 Recommendations were published as Publication 60 [62]. ICRP Supporting Guidance 1 [63] examined the risks associated with exposure to radiation as an evidential foundation for Publication 60. The results from the RERF studies of the Japanese atomic bomb survivors published since the 1977 Recommendations were reviewed. Longer follow-up of the LSS cohort and the adoption of the DS86 doses led to a ‘lifetime risk of fatal cancer for a member of the general population exposed to low-level whole-body irradiation [that] can be assumed to average ∼5% per Sv, thus exceeding that estimated in ICRP [Publication] 26 by a factor of about 3–4’ [63]. This notable change in the risk estimate used by ICRP was based upon the recent RERF studies of the atomic bomb survivors, and led to reductions in annual effective dose limits to 20 mSv for workers and 1 mSv for members of the public, dose limits that still apply today.

The publication of the RERF cancer incidence studies in 1994 [64–67], the first comprehensive examination of the registration data covering both solid cancers [65] and leukaemia [66], marked an important step forward in the study of cancer risks in the survivors, particularly for those cancer types with low lethality, such as thyroid cancer. The (subsequently updated) cancer incidence data were to play the leading role in the quantitative cancer risk evaluation used in the ICRP 2007 Recommendations [1].

LSS Report 12 updated cancer mortality in 1996 [68] and non-cancer mortality in 1999 [69], but just into the new millennium Dosimetry System 2002 (DS02) [70] was introduced. LSS Report 13 (2003) [71] and associated studies [72, 73], and Preston *et al*. [73], examined the impact on risk estimates of the adoption of the DS02 doses. Individual DS02 dose estimates were ‘not greatly different’ from DS86 estimates [70] and the introduction of the DS02 doses did not lead to any major changes in cancer risk estimates [73].

The updated RERF study of solid cancer incidence, using the DS02 doses, was published in 2007 [74] and played a fundamental role in the nominal cancer risk coefficients derived in the next set of ICRP Recommendations. The ICRP 2007 Recommendations, published as Publication 103 [1], relied heavily on the LSS cancer incidence data, in particular, solid cancer incidence data for 1958–98 [74] and leukaemia incidence data for 1950–2000 [75]. excess relative risk (ERR) and excess absolute risk (EAR) models were developed from these incidence data for 11 cancer types: cancers of the oesophagus, stomach, colon, liver, lung, female breast, ovary, bladder, thyroid and all other solid cancer combined (excluding cancers of the skin and bone, for which the nominal risk coefficients of Publication 60 were used) and leukaemia. The solid cancer models were linear in dose (with a minimum latent period of 5 years) and modified by sex, age-at-exposure and attained age, while the leukaemia models were linear-quadratic in dose (with a minimum latent period of 5 years) and modified by sex, age-at-exposure and time-since-exposure [75]. Clearly, the Japanese survivor data were key in generating the nominal lifetime risks of cancer incidence per organ/tissue dose derived in the ICRP 2007 Recommendations [1].

Subsequent to ICRP Publication 103, LSS Report 14 [76] updated mortality among the survivors to end-2003, and Hsu *et al*. [77] updated the incidence of leukaemia (and other lympho-haematopoietic malignancies) to end-2001. Then in 2017, doses were revised again with the introduction of Revision 1 of the Dosimetry System 2002 to produce DS02R1 doses [78]. Grant *et al*. [79] updated the cancer incidence analyses using DS02R1 doses with follow-up to end-2009 and adjustment for smoking. An elevation in the sex-averaged linear ERR was detectable in the 0–100 mGy dose range. They found differences in the variation of the ERR with dose for all solid cancers combined between males and females: although for females the dose-response was linear, for males upward curvature was evident. As Grant *et al*. noted [79], ‘this analysis demonstrates that solid cancer risks remain elevated more than 60 years after exposure’.

**Figure 1:**
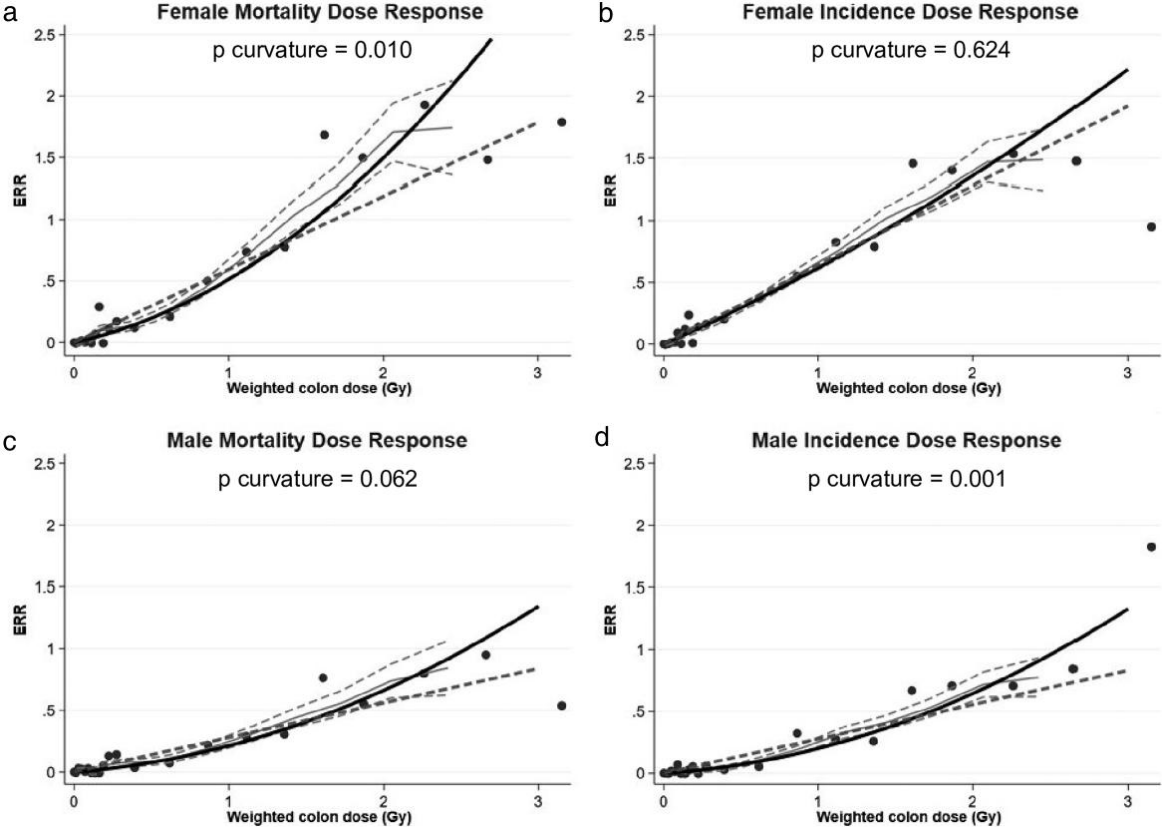
Variation of the excess relative risk (ERR) of all solid cancers combined with DS02R1 weighted colon absorbed dose, Life Span Study (1958–2009), for (a) female mortality, (b) female incidence, (c) male mortality, and (d) male incidence. Categorial points (black circles), fitted linear-quadratic (thick-black solid lines) and linear dose-response functions (thick-grey dashed lines), and non-parametric smoothed estimates (thin-grey solid lines) with pointwise 95% confidence intervals (thin-grey dashed lines). The ERRs are shown for subjects at attained age 70 years after exposure at age 30 years. *P* curvature is the *P-*value for quadratic departure from linearity. Adapted from Fig. 2 of Brenner *et al*. [80]

Brenner *et al*. [80] used cancer incidence and mortality data for follow-up during 1958–2009 and DS02R1 doses to compare solid cancer dose-responses for males and females, mortality and incidence, and found important differences: the shapes of the sex-specific dose-responses for incidence found by Grant *et al*. [79] were confirmed, while for mortality there was evidence of upward curvature for females and an indication of upward curvature for males [80], see Fig. 1. The reasons for these patterns are the subject of continued study at RERF, but are not attributable to the change from DS02 to DS02R1 doses [80]. They are likely to be related to the different contributions from the various sites of cancer, so it is apparent that a rather complex mixture of cancer-site-specific dose-responses are present in the latest LSS data.

A number of papers have been published in recent years addressing particular types of cancer and deriving models based on incidence data to end-2009. These models are of substantial interest for radiological protection because, *inter alia*, information on sites of cancer that were not considered separately in the modelling of ICRP Publication 103 have been produced, such as for central nervous system cancers [81], and evidence has strengthened for types of cancer for which an association with irradiation has previously been absent or weak, such as prostate cancer [82] and pancreatic cancer [83]. For cancers of the female breast [84] and uterine corpus [85], the identification of a window of sensitivity to radiation exposure during puberty sheds new light on how to take account of the modifying effect of age when modelling the dose-risk relationships.

These results illustrate how findings from the follow-up of the atomic bomb survivors are developing 80 years after exposure, and these findings will have to be accommodated in the ICRP system at some suitable point. The recent RERF studies address cancers that are incident decades after exposure among those exposed at a young age, and Grant *et al*. reported that of the ∼112 000 members of the LSS solid cancer incidence cohort, ∼38% were still alive at the end of 2009 so there is substantial information still to come [79]. This emphasizes the importance of RERF continuing to collect, collate and analyse data from the Japanese atomic bomb survivors to obtain a comprehensive understanding of the risk by cancer type in terms of modification by sex, age-at-exposure and time-since-exposure/attained age. This is vital for the system of radiological protection. The continuing flow of information from the RERF studies has been recognized by ICRP by, for example, the setting up of Task Group 122 (https://www.icrp.org/icrp_group.asp?id=196), among other work preparing for the next set of Recommendations.

### 3.4. Intrauterine exposure

As noted above, as early as 1952 there was a report of microcephaly and mental retardation among those who had been *in utero* at the time of the Hiroshima bombing and relatively close to the hypocentre [33], and small head size and mental retardation among the survivors heavily exposed *in utero* was confirmed by later studies [63, 86–92]. The early study of Miller [86] was employed in the somatic risk evaluation reported in ICRP Publication 8 [21, 22]. Severe mental retardation, reduction in intelligence quotient (IQ), school performance, and seizure disorders among the intrauterine exposed survivors were the subjects of study in later reports [89]. ICRP Publication 90 [93] recognized the Japanese atomic bomb survivors irradiated *in utero* as the ‘most informative’ as far as mental development is concerned, and the ABCC/RERF studies formed the basis of protection against such effects in the ICRP 2007 Recommendations [1]: for severe mental retardation, a dose threshold of 300 mGy was assumed and that if there were to be any reduction in IQ for doses <100 mGy then it would be ‘of no practical significance’ [1].

A number of publications, most notably those from the Oxford Survey of Childhood Cancers (OSCC) [94], had suggested that the risk of childhood cancer was associated with a medical abdominal x-ray examination of the pregnant mother. However, Jablon and Kato [95] reported that only one cancer death had occurred in childhood among 1292 Japanese atomic bomb survivors irradiated *in utero*, and that this was not a case of leukaemia, which was much less than expected on the basis of the findings of the OSCC. This led to a long-running exchange of opinions on the risk of childhood cancer posed by intrauterine exposure to radiation.

Mole [96] made the prescient observation (see below) that when Jablon and Kato [95] had found a discrepancy between the number of childhood cancer deaths observed in the bomb survivors irradiated *in utero* and the number predicted on the basis of the findings of antenatal x-ray examinations, ‘the basic assumptions underlying these comparisons may be in error’. Mole suggested that a raised sensitivity to cell killing *in utero* together the higher doses received during the bombings may reduce the excess risk of cancer among the survivors. The problems with such comparisons have also been emphasized by others [97–99].

The ICRP 2007 Recommendations concluded from the evidence available at the time:

‘The Commission considers that it is prudent to assume that life-time cancer risk following in-utero exposure will be similar to that following irradiation in early childhood, i.e. at most, about three times that of the population as a whole’.

**Figure 2:**
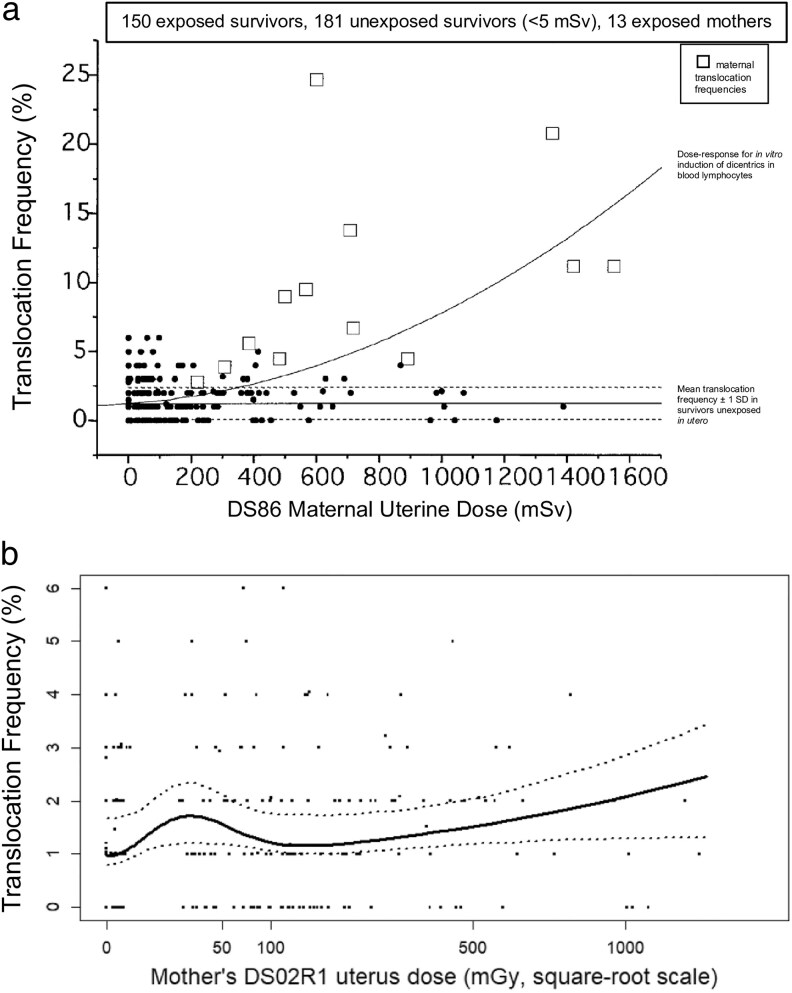
The variation with maternal uterine dose of chromosome translocation frequency in peripheral blood lymphocytes sampled at around 40 years of age from Japanese survivors *in utero* at the time of the bombings, (a) using DS86 doses (and also showing translocation frequencies for 13 exposed mothers) [100], and (b) using DS02R1 doses [101]. Figure 2(a) is adapted from Fig. 1 of Ohtaki *et al*. [100], and Fig. 2(b) is Fig. 4 of Cologne et al. [101]. Figure 2(a) is reproduced with permission from [100] (^©^ 2025 Radiation Research Society)

A remarkable finding from the atomic bomb survivors who were irradiated *in utero* was reported by Ohtaki *et al*. [100], who analysed the frequency of stable chromosome translocations in peripheral blood lymphocytes sampled at around 40 years of age from 331 persons *in utero* at the time of the bombings, 150 of whom were within 2 km of the hypocentre. The variation of the translocation frequency with DS86 maternal uterine dose is shown in Fig. 2a, and that found by a later analysis of Cologne *et al*. [101] in terms of DS02R1 maternal uterine dose is shown in Fig. 2b. Surprisingly, in contrast to the expected level of increase of translocation frequency with dose found in experimental studies and in mothers of 13 of the *in utero* exposed survivors (Fig. 2a), there was hardly any evidence for an increase in translocation frequency at doses >100 mGy. There was, however, some evidence for an increase at doses <100 mGy.

Ohtaki *et al*. [100] interpret this finding as indicating that a proportion of lymphoid precursor cells present during intrauterine development are particularly sensitive to chromosome damage but also to such damage resulting in cell death, and that at doses approaching 100 mGy and above cell killing eliminates these damaged cells. This has clear implications for the risk of leukaemia following the receipt of moderate and high doses received *in utero* and potentially explains the absence of cases of childhood leukaemia among the Japanese bomb survivors irradiated *in utero*—an echo of Mole’s observation back in 1974 [96]. The question remains as to whether this effect persists for any time after birth, and if so, for how long. In this respect, of interest is the observation of Delongchamp *et al*. [98] that no case of leukaemia was found among the Japanese bomb survivors exposed during the first 9 months after birth, although the small number of such births would preclude any reliable inferences. The evidence from the chromosome study of Japanese survivors irradiated *in utero* is manifestly of importance to the understanding of the risks of intrauterine exposure to radiation, and potentially to other exposures *in utero* and to the mechanistic basis of leukaemia.

The cohort of atomic bomb survivors irradiated *in utero* represents one of the few opportunities for investigating the risk of adult cancer following intrauterine exposure, and is the most informative source of data for doing so. Recent RERF studies have found positive dose-responses for solid cancers for both incidence [102] and mortality [103], although the mortality dose-response was positive for females only. Of note is that of the 908 survivors who had received an intrauterine dose >5 mGy, only 15% had died by the end of follow-up (2012) [103], so substantial evidence remains to be derived from these survivors, which will undoubtedly inform radiological protection.

These recent findings for the survivors irradiated *in utero* will clearly need to be considered in the next set of ICRP Recommendations, and is one of the areas being covered by ICRP Task Group 121 (https://www.icrp.org/icrp_group.asp?id=189).

### 3.5. Long-term non-cancer diseases

In LSS Report 7 (1982) [54], a separate study was conducted of non-cancer disease mortality, without finding evidence of a relationship with radiation dose. However, 10 years later, LSS Report 9 [59] re-examined non-cancer mortality, using DS86 doses and an extended follow-up, and found an excess of non-cancer deaths among survivors receiving doses >2 Gy while <40 years of age, which was due to circulatory and digestive diseases, although the relative risk was much smaller than that for cancer. LSS Report 12 (1999) [69] found dose-related increases in mortality from circulatory, digestive and respiratory diseases, with rates raised by ∼10% at 1 Sv, which is much smaller than that for cancer, a pattern of results repeated in LSS Report 13 (2003) [71], with positive dose-responses reported for circulatory, digestive and respiratory diseases. ICRP, in its 2007 Recommendations [1], concluded that the ‘available data on possible excess in non-cancer diseases (e.g. cardiovascular disorders) are judged to be insufficient to inform on risks at low doses’.

Specific attention was given to mortality from diseases of the circulatory system in the atomic bomb survivors by the study of Shimizu *et al*. [104] in 2010. Heart disease and stroke were considered separately with linear ERR/Gy estimates of 0.14 (95% CI: 0.06, 0.23) and 0.09 (95% CI: 0.01, 0.17), respectively, but with some indication of upward curvature in the dose-response for stroke. This prompted considerable discussion on how diseases of the circulatory system should be treated by ICRP. As a result, in 2012, ICRP issued a Statement [105] noting that the ‘absorbed dose threshold for circulatory disease may be as low as 0.5 Gy to the heart or brain’.

Studies of the survivors have continued to provide evidence for informed discussions on whether diseases of the circulatory system need to be included in the scheme of protection against low levels of exposure [106–111]. In this respect, ICRP has established Task Group 119 (https://www.icrp.org/icrp_group.asp?id=185) to examine the evidence.

One of the first reports of health effects in the survivors was of cataracts in those heavily exposed (see above), and ocular opacity studies have continued. That of Neriishi *et al*. [112, 113] was instrumental in persuading ICRP that the dose threshold for cataract formation should be reduced from 5 Gy [1] to 0.5 Gy in its 2012 Statement [105]; the annual dose limit for the lens of the eye was accordingly reduced to 20 mSv. New RERF analyses of survivor data are ongoing that should help in clarifying radiation effects for different subtypes of cataract.

## 4. Concluding remarks

No single dataset or study can completely meet all the requirements of an international system of radiological protection. The studies of the atomic bomb survivors are of a mid-20th century Japanese population that had suffered the privations of a long war, was exposed to a single brief burst of gamma (and some neutron) radiation, with many of the healthy men away on military service, and to enter the LSS cohort in October 1950 survivors had to have remained alive throughout the difficult 5 years following the bombings. So, for example, the experience of the survivors does not directly address the risks in a healthy workforce receiving many small doses of radiation received at a low dose rate, or those from continual inhalation of the alpha-particle emitting radioisotopes of radon and their radioactive decay products present in the home.

A large number of epidemiological studies have now been carried out on various exposure situations (occupational, medical or environmental exposures) and different populations [114–117]. These results complement those provided by the ABCC/RERF studies of the cohort of survivors of the atomic bombings of Hiroshima and Nagasaki [118]. The synthesis of all these results will enhance our knowledge of the health effects of ionising radiation and help to consolidate the radiological protection system.

But in that framework, it is notable that the studies of the atomic bomb survivors, given the care and rigour with which they have been conducted, have provided a solid foundation for the international system of radiological protection to a greater extent than any other study. This remains the case: the information provided by the ABCC/RERF studies contributes to all the Task Groups currently implemented by the ICRP Committee 1 to improve the assessment of irradiation-related health effects in humans (https://www.icrp.org/icrp_group.asp?id=7). These results are fundamental to quantifying the long-term risks of exposure to radiation, and the modification of these risks by sex, age-at-exposure and time-since-exposure/attained age. They continue to provide substantial information on the increased risks of cancer, on the characterization of the slope of the dose-risk relationship and on the assessment of the impact of the dose rate on this relationship. Further, the data represent a major source of information for estimating the relative biological effectiveness of high-energy gamma and neutron radiations. Beyond cancer, the survivors provide material evidence on various non-cancer diseases many decades after exposure and on risks in the descendants of those irradiated. For all these reasons, the RERF studies of the atomic bomb survivors continue to provide results that are invaluable in consolidating the system of radiological protection. This is particularly true today in the process of reviewing and revising the radiological protection system, launched by the ICRP in respect of its next general recommendations [119–122].

## Data Availability

Only publicly available and published data are used.
